# Standard and accelerated crosslinking protocols in keratoconus - differences and evolution at one year

**DOI:** 10.22336/rjo.2025.29

**Published:** 2025

**Authors:** Maria-Silvia Dina, Mihaela-Monica Constantin, Maria-Cristina Marinescu, Cătălina-Gabriela Corbu, Cătălina-Ioana Tătaru, Călin-Petru Tătaru

**Affiliations:** 1 Doctoral School, “Carol Davila” University of Medicine and Pharmacy, Bucharest, Romania; 2 Oftaclinic Ophthalmology Clinic, Bucharest, Romania; 3 Ophthalmology Department, “Carol Davila” University of Medicine and Pharmacy, Bucharest, Romania

**Keywords:** keratoconus, corneal crosslinking, standard protocol, accelerated protocol, corneal biomechanics, CXL = Corneal collagen crosslinking, CH = Corneal hysteresis, CRF = Corneal resistance factor, IOPg = Goldmann-correlated intraocular pressure, IOPcc = Cornea-correlated IOP, KC = Keratoconus

## Abstract

**Objectives:**

Keratoconus (KC) is a bilateral, progressive corneal ectasia that involves corneal thinning and a decrease in visual acuity. Stopping the progression of keratoconus can be achieved through various photooxidative crosslinking (CXL) methods. The objective of this study was to compare two protocols of epi-off corneal crosslinking—the standard and the accelerated protocol—in terms of efficacy after a one-year follow-up.

**Methods:**

41 eyes with progressive keratoconus were treated with corneal crosslinking, either using the accelerated (Acc-CXL) or standard protocol (Std-CXL). The following parameters were monitored: refraction, corneal diopter power on topographic maps (Kmax and Kmin), corneal thickness (CCT), resistance factor (CRF), hysteresis, and the depth of the demarcation line. All measurements were repeated 12 months after the intervention.

**Results:**

The progression of keratoconus was halted in 20 eyes using the accelerated method and in 21 eyes using the standard procedure. Both methods resulted in a statistically significant regression of the spherical equivalent, Kmax, and an increase in CCT and CRF, without substantial differences in efficacy. The demarcation line was highlighted on average at a depth of 278.9 ± 31.71 micrometres for the Acc-CXL group and 280.42 ± 47.85 micrometres for the Std-CXL group. It was correlated with the initial topographical values.

**Discussion:**

The evaluation of patients revealed no progression of keratoconus following the procedure. Approximately 40% of the cases in the accelerated protocol group and 38.09% of the cases in the standard protocol group have maintained the parameters at a constant level. In comparison, approximately 60% of the cases have shown improvements. An Australian registry revealed that both CXL protocols are safe and effective; however, the standard procedure leads to improved visual acuity, a more significant flattening of the steepest meridian, and a higher chance of an effect greater than one diopter power.

**Conclusions:**

Corneal crosslinking (CXL) was effective in halting the progression of keratoconus using both methods. Accelerated CXL is faster and more comfortable for patients, with similar efficiency to standard CXL.

## Introduction

Keratoconus (KC) is a bilateral, progressive corneal ectasia characterized by corneal thinning, which leads to an increase in myopic astigmatism and a decrease in visual acuity [1,2]. Research has not identified the etiopathogenesis of this condition with certainty; however, several risk factors are known to be involved, including eye rubbing, family history of keratoconus, and atopic backgrounds such as allergy, asthma, eczema, and disorders of the endocrine system [3]. The natural evolution of the disease involves a higher incidence in adolescence and between the ages of 20 and 35 years, followed by a lower incidence and slower progression after the age of 40 [4].

Nowadays, we can halt the progression of keratoconus by performing photooxidative crosslinking, which is crucial for preserving patients’ visual function and preventing complications associated with keratoconus. The first CXL protocol was first applied to keratoconus patients in the early 2000s and involves the removal of the corneal epithelium (thus the first protocol was called “epi-off”), after which the corneal stroma is saturated with riboflavin (vitamin B2) for 30 minutes and then, ultraviolet A light (wavelength of 370 nm) is applied for 30 minutes. Most commonly, after removal of the corneal epithelium, patients experience pain and ocular discomfort for several days following the procedure. Additionally, 3-8% of patients may experience delayed epithelial healing. To avoid these disadvantages, various modifications have been made to the standard protocol. Reducing the exposure time or increasing the intensity of UVA exposure (accelerated or customized CXL), modifying the composition of the riboflavin solution by using excipients that promote better stromal penetration through an intact epithelium (epithelial technique), and iontophoresis are possible options [5].

The Dresden protocol has been established as the gold standard for the procedure [6]. Various variants of accelerated procedures have been created, based on the Bunsen-Roscoe reciprocal law: “a photochemical reaction should remain constant if the total energy delivered is kept constant” (the setting corresponds to this fluence of 5.4 J/cm2) [6].

Besides UVA light, riboflavin is an essential element in the crosslinking procedure. This molecule has a central role in the crosslinking process, acting as a photosensitizer to produce oxygen singlets and riboflavin triplets. These free radicals interact with the proteins of the corneal stroma and are responsible for changing its biomechanical properties [7]. Furthermore, riboflavin absorbs the majority of UVA radiation within the anterior stroma, thereby preventing potential damage to ocular structures, such as the corneal endothelium, lens, and retina [8]. The efficacy of CXL is dependent on adequate stromal riboflavin uptake, as the corneal epithelium has sufficient permeability for lipophilic molecules with a molecular weight of less than 180 Da [5]. In comparison, riboflavin is a large hydrophilic molecule with a molecular weight of 340 Da [9]. Epithelial removal determines the loss of epithelial tight junctions, permitting sufficient and homogeneous stromal uptake of riboflavin [10]. Riboflavin concentration decreases with increasing depth and increases with longer application time. Measuring the fluorescence, it was proven that after 30 minutes of riboflavin application at the surface of the cornea (epi-off), there is an almost linear decrease in riboflavin concentration from the anterior to the posterior stroma [11].

Thus, CXL aims to stabilize corneal ectasia. This surgical procedure increases the biomechanical strength of the cornea by forming chemical bonds between collagen fibrils, thereby inhibiting the progression of keratoconus. The transition from cross-linked to non-cross-linked tissue is visible on biomicroscopy as an anterior stromal zone with higher reflectivity, caused by an area of honeycomb edema and keratocyte apoptosis. This can also be demonstrated by confocal microscopy to a depth of approximately 300 μm. A clear demarcation line, a sharp transition from cross-linked to non-cross-linked stroma, can be detected by OCT. However, both imaging methods, OCT and confocal microscopy, have been used to assess the corneal stroma demarcation line in recent studies. The depth of the demarcation line is a simple and valuable tool for clinicians to determine the depth of the cross-linking effect [11-13].

The objective of this study was to investigate the evolution of biomechanical, topographical, and refractive properties of the cornea in keratoconus patients, before and after undergoing corneal collagen crosslinking, by comparing the standard and accelerated protocols.

## Materials and methods

This retrospective study evaluates the evolution of keratoconus cases one year after accelerated or standard CXL was performed using the epi-off method. All patients who presented at the Oftaclinic Clinic in Bucharest between 2021 and 2022 were screened for inclusion criteria. Patients and their legal representatives (in the case of minors) offered informed consent. The Oftaclinic Ethics Committee approved the study.

All patients underwent a complete ocular consultation, which also included records of refraction (using auto-refractometer records), maximum and minimum corneal dioptres measured with Aladdin corneal topography (Topcon, Tokyo, Japan), demarcation line depth, measured with Optical Coherence Tomography (OCT), central corneal thickness (CCT), corneal biomechanical properties: corneal hysteresis (CH) and corneal resistance factor (CRF), measured with Ocular Response Analyzer (ORA) (Reichert Ophthalmic Instruments Inc, Depew, NY, USA).

Patients were included if they met criteria for progressive keratoconus: increase of corneal diopter on the steepest meridian (Kmax) more than 1 D, increase of average of corneal diopter more than 0.75 D, increase of spherical equivalent (SE) more than 0.50 D, thinning of corneal thickness more than 5% or more than 20 microns, history of decreased visual acuity.

Exclusion criteria were represented by corneal thickness less than 400 microns, history of keratitis or corneal surgery, pregnancy, and corneal scars.

The Amsler Krumeich classification was used to stage the keratoconus cases, a system that accounts for the degree of myopia and astigmatism, mean keratometry, and minimal central corneal thickness [12,13].

The data analyzed in this study were obtained from measurements performed before the CXL intervention and 12 months after the intervention. All patients underwent the epi-off procedure, performed in a sterile operating room under topical anesthesia, which involved debridement of the corneal epithelium, instillation of a 0.1% riboflavin solution, and exposure to ultraviolet A light for a total energy dose of 5.4 Joules/cm^2^ [1]. However, one cohort of patients underwent the accelerated procedure, which involves soaking the cornea in a riboflavin solution for 20 minutes, followed by exposure to a UVA intensity of 9 mW/cm^2^ for 10 minutes. The other cohort of patients underwent the standard procedure, which involved soaking the cornea in a riboflavin solution for 30 minutes, followed by exposure to a UVA intensity of 3 mW/cm^2^ for 30 minutes [14]. An experienced surgeon performed all procedures, and the choice of procedures was not randomized but was decided by the surgeon based on the clinical characteristics of each patient. After the intervention, patients followed a topical treatment with broad-spectrum antibiotics and non-steroidal anti-inflammatory drops, and a soft contact lens was placed until corneal reepithelialization occurred.

To identify significant differences between time points of the same patient (before and after CXL), Levene’s Test was followed by a dependent t-test. To identify significant differences between the accelerated and standard procedures, Levene’s Test was followed by an independent t-test. The degree of correlation between variables was calculated using Pearson’s correlation coefficient (“Pearson’s r”). Correlations were deemed weak (Pearson’s r between 0.3 and -0.3), moderate (Pearson’s r between 0.3 and 0.5 or -0.3 and -0.5), and strong (Pearson’s r over 0.5, under -0.5). The p-value of 0.05 was considered the threshold for statistical significance. Statistical analysis was performed using the Statistical Package for the Social Sciences (SPSS), version 26 (IBM Corp., Armonk, NY, USA).

## Results

### Descriptive results

The accelerated CXL cohort consisted of 20 eyes, comprising 15.00% females and 85.00% males, with an average age of 24.55 ± 6.27 years. According to the Amsler-Krumeich criteria, two eyes were diagnosed with stage I keratoconus, ten eyes with stage II, two eyes with stage III, and six eyes with stage IV.

The standard CXL cohort consisted of 21 eyes, comprising 33.33% females and 66.67% males, with an average age of 19.80 ± 5.99 years. According to the Amsler-Krumeich criteria, three eyes were diagnosed with stage I of keratoconus, nine eyes with stage II, two eyes with stage III, and seven eyes with stage IV.

The two cohorts had similar baseline characteristics (**Table 1, 2**). However, the age was significantly lower (p value 0.018), and the refractive cylindrical value was considerably higher in the standard procedure group (p value 0.026).

**Table 1:** Descriptive statistics of the accelerated CXL cohort, before and after corneal collagen crosslinking

Accelerated protocol		Mean	Std. Deviation	Mean difference	Paired samples T test p value
Sphere (D)	Before CXL	-4.163	2.916	-0.438	0.119
	After CXL	-3.725	2.580		
Cylinder (D)*	Before CXL	-4.113	2.218	-0.638	0.007
	After CXL	-3.475	2.489		
Spherical equivalent (D)*	Before CXL	-6.219	3.081	-0.756	0.023
	After CXL	-5.463	2.522		
Kmax (D)*	Before CXL	53.497	4.860	1.276	0.001
	After CXL	52.221	4.124		
Kmin (D)	Before CXL	47.953	4.077	0.237	0.556
	After CXL	47.716	3.186		
CCT (micrometers)*	Before CXL	456.050	39.656	-5.650	0.001
	After CXL	461.700	37.311		
CH (mmHg)	Before CXL	8.760	1.837	-0.375	0.089
	After CXL	9.135	2.185		
CRF (mmHg)*	Before CXL	7.585	2.170	-0.505	0.024
	After CXL	8.090	2.278		
Demarcation line absolute depth (micrometers)	After CXL	278.900	31.716		
Demarcation line depth relative to CCT (%)	After CXL	61.21%	5.02%		

* denotes a statistically significant difference after CXL

**Table 2:** Descriptive statistics of the standard CXL cohort, before and after corneal collagen crosslinking

Standard protocol		Mean	Std. Deviation	Mean difference	Paired samples T-test p-value
Sphere (D)	Before CXL	-3.679	3.791	-0.369	0.216
	After CXL	-3.310	3.859		
Cylinder (D)*	Before CXL	-6.119	3.200	-0.952	0.008
	After CXL	-5.167	2.566		
Spherical equivalent (D)*	Before CXL	-6.738	4.024	-0.845	0.027
	After CXL	-5.893	3.773		
Kmax (D)*	Before CXL	53.980	5.408	1.462	0.001
	After CXL	52.518	4.846		
Kmin (D)	Before CXL	47.182	3.992	0.331	0.185
	After CXL	46.851	3.790		
CCT (micrometers)*	Before CXL	466.095	39.919	-3.286	0.037
	After CXL	469.381	41.333		
CH (mmHg)	Before CXL	8.491	1.478	-0.286	0.067
	After CXL	8.776	1.515		
CRF (mmHg)*	Before CXL	7.433	1.281	-0.414	0.048
	After CXL	7.848	1.200		
Demarcation line absolute depth (micrometers)	After CXL	280.429	47.857		
Demarcation line depth relative to CCT (%)	After CXL	60.04%	7.94%		

* denotes a statistically significant difference after CXL

The accelerated crosslinking protocol led to significant improvements (**Table 1**). After CXL, the cylindrical refractive value, spherical equivalent, and Kmax were significantly lowered, and CCT and CRF were substantially higher.

Similar results were found in the standard crosslinking protocol group (**Table 2**). After CXL, the cylindrical refractive value, spherical equivalent, and Kmax were significantly lowered, and CCT and CRF were substantially higher.

When comparing the two protocols, both were proven to be similarly efficacious. There were no statistically significant differences in the magnitude of improvement in SE, CCT, maximum and minimum keratometry, CH, CRF, or the depth of the demarcation line (**Table 3**).

**Table 3:** Average difference of variables, before and after crosslinking, in the Accelerated (Acc CXL) and Standard (Std CXL) protocol groups

	Study group	Mean difference	Std. Deviation	Levene’s Test for Equality of Variances	Independent t-test p-value
SE difference before and after CXL	Acc CXL	0.756	1.365	Equal variances assumed	0.850
	Std CXL	0.845	1.619		
CCT difference before and after CXL	Acc CXL	5.650	6.523	Equal variances assumed	0.261
	Std CXL	3.286	6.732		
Kmax difference before and after CXL	Acc CXL	-1.276	1.428	Equal variances assumed	0.710
	Std CXL	-1.462	1.737		
Kmin difference before and after CXL	Acc CXL	-0.237	1.770	Equal variances assumed	0.839
	Std CXL	-0.331	1.105		
CH difference before and after CXL	Acc CXL	0.375	0.934	Equal variances assumed	0.727
	Std CXL	0.286	0.677		
CRF difference before and after CXL	Acc CXL	0.505	0.919	Equal variances assumed	0.751
	Std CXL	0.414	0.901		

SE = spherical equivalent, CCT = central corneal thickness, Kmax = maximum keratometry, Kmin = minimum keratometry, CH = corneal hysteresis, CRF = corneal resistance factor

Importantly, no patients experienced a Kmax increase of more than one diopter following the intervention (**Table 4**). A similar proportion of patients had a stable Kmax (changes +1D to -1D) in the two groups (40.0% for Accelerated-CXL and 38.09% for Standard-CXL).

**Table 4:** Proportion of patients in each study group and the degree of improvement in terms of Kmax flattening

Variation of Kmax	Accelerated CXL (n = 20)	Standard CXL (n = 21)
> + 1D	0	0
+ 1D to - 1D	40.0%	38.09%
- 1D	30.0%	47.63%
- 2D	5.0%	9.52%
- 3D	25.0%	4.76%

**Fig. 1:**
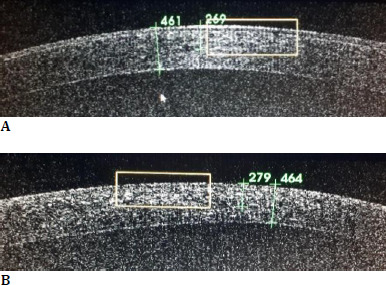
OCT aspect of the demarcation line and the depth reported to the total thickness of the cornea, after the accelerated protocol (A) and after the standard protocol (B)

The demarcation line was identified using Anterior Segment Optical Coherence Tomography (OCT), and the depth was reported as a percentage of the total corneal thickness. The average absolute depth was at 278.90 micrometers in the accelerated group and at 280.43 micrometers in the standard group. The average depth relative to the corneal thickness was 61.21% in the accelerated group and 60.04% in the standard group. The two groups did not differ significantly in terms of the depth of the demarcation line (p-value = 0.904) or regarding the relative depth (p-value = 0.574) (**Fig. 1A, B**).

**Fig. 2:**
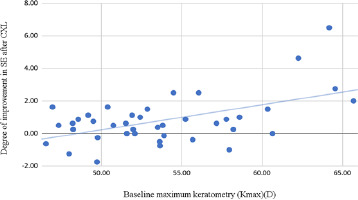
Correlation between the baseline Kmax in the entire cohort and the improvement in SE after the CXL procedure

There were several significant correlations between the differences before and after the procedure and the baseline characteristics of the patients. The degree of refractive improvement (i.e., the decrease in SE) was higher for patients who were more myopic and astigmatic, showing a negative correlation with baseline sphere (r = -0.332, p = 0.034) and with baseline cylinder (r = -0.319, p = 0.042). The steeper the initial corneal shape was (positive correlations with Kmax, r = 0.529, p < 0.001, and with Kmin, r = 0.465, p = 0.002) (**Fig. 2**).

**Fig. 3:**
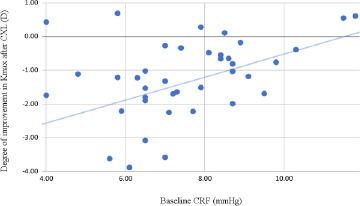
Correlation between the baseline CRF in the entire cohort and the improvement in Kmax after the CXL procedure

The biomechanical improvement of CRF increase was correlated positively with the cylindrical values (r 0.326, p value 0.038) (i.e., the lower the cylindrical error, the better the CRF improvement). The topographical improvement of Kmax was also positively correlated with the baseline cylindrical value (r 0.327, p value 0.037), SE (r 0.417, p value 0.007) and CRF (r 0.378, p value 0.015), and negatively correlated with baseline Kmin (r -0.515, p value 0.001) and Kmax (r -0.532, p value <0.001) - i.e. the steeper the cornea was. The higher the astigmatism before the procedure, the more the Kmax flattens after the procedure (**Fig. 3**).

**Fig. 4:**
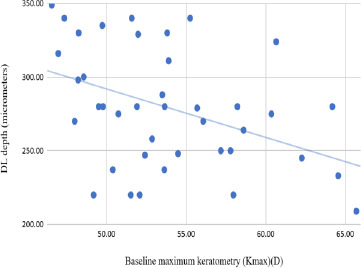
Correlation between the baseline Kmax in the entire cohort and the depth of the demarcation line after the CXL procedure

The absolute demarcation line depth had a negative correlation both with Kmax (r=-0.416, p value 0.007) and with Kmin (r=-0.314, p value 0.045) (i.e., the DL was deeper the flatter the cornea was) (**Fig. 4**).

## Discussion

In this study, we demonstrated the efficacy of corneal collagen crosslinking in halting and even reversing the progression of keratoconus. By applying either the accelerated or the standard protocol, statistically significant improvements are obtained in several essential parameters.

Numerous studies have demonstrated the efficacy and safety of the standard CXL protocol; however, to mitigate the discomfort associated with prolonged intervention, the epi-off accelerated CXL protocol was introduced into clinical practice [8]. This requires a change in the riboflavin solution composition to enhance the stromal penetrance. It is well known that the photooxidative crosslinking and its safety depend on the amount of riboflavin in the corneal stroma. In the present study, we used a riboflavin solution containing hypromellose, which facilitates the diffusion of the active substance into the stromal layer. The selection of patients for either the standard or the accelerated procedure is based on their age. It is well established that disease progression is more likely in young patients, and studies have demonstrated the efficacy of the standard method [15].

Some authors recommend accelerated CXL for children, as the procedure is quicker, while others suggest that more extended follow-up periods may reveal diverging evolutions of the two groups [8]. As such, the efficacy of the two epi-off procedures was compared, and a 2020 study found that after 2 years of follow-up, children who underwent the accelerated procedure had a worse outcome than those who underwent the standard procedure, in terms of refraction, topography, and visual acuity [16]. A recent meta-analysis compared the two protocols used in paediatric keratoconus and found no difference in terms of Kmax, SE, and CCT, but with a better evolution of standard CXL in terms of visual acuity after two years [17]. Therefore, in the present study, the two protocol groups differed significantly: the patients in the standard group were substantially younger. They had significantly higher astigmatism, as the standard procedure was preferred in young paediatric patients.

Importantly, in the present cohort, there were no differences in the depth of the demarcation line (as a percentage of the entire corneal thickness), which has been suggested as a surrogate indicator of efficacy [8]. Therefore, our results indicate that the efficacy of standard and accelerated corneal crosslinking is comparable in this cohort.

In the present study, both protocols were effective in halting the progression of corneal ectasia and even reversing refractive, pachymetry, topographic, and biomechanical parameters after one year. When studying the two groups individually, similar results were found. The spherical, cylindrical refractive values and maximum keratometry were decreased, and CH and CRF were increased.

As presented in **Table 3**, the evaluation of patients revealed no progression of keratoconus following the procedure. Approximately 40% of the cases in the accelerated protocol group and 38.09% of the cases in the standard protocol group have maintained the parameters at a constant level. In comparison, approximately 60% of the cases have shown improvements. An Australian registry revealed that both CXL protocols are safe and effective; however, the standard procedure leads to improved visual acuity, a more significant flattening of the steepest meridian, and a higher chance of an effect greater than one diopter power [18].

Similar results to ours were obtained in the literature by comparing standard and accelerated epi-off CXL procedures. In a two-year follow-up of a Romanian cohort, visual acuity, refraction, corneal thickness, and the steepest corneal meridian all showed similar improvements, with minor differences noted. Specifically, BCVA improved more quickly in the accelerated group, while in the standard group, a deeper demarcation line was achieved [19].

In our study, a decrease in cylindrical refractive error and spherical equivalent was observed, along with a reduction in maximum keratometry. This change was larger in the standard group (Kmax decreased on average by 1.462 D, compared to 1.276 D in the accelerated group), which may be explained by the younger patients in the standard group, still undergoing growth and hormonal fluctuations [20].

As the CXL procedure strengthens the links between collagen fibrils in the corneal stroma, it has been suggested in the literature that the biomechanical properties of the cornea should improve after the procedure [21]. Preclinical studies involving ex vivo human corneas demonstrate that the rigidity is more than three times greater [22], and the diameter of collagen fibers increases following crosslinking in animal models [23].

Two devices are available for investigating corneal biomechanics: Corvis ST and ORA [21]. The latter was used in the present study, and the two parameters (corneal hysteresis and resistance factor) were significantly increased after both the standard and the accelerated CXL, which is in line with previous reports in the literature [24]. On the other hand, other studies have not found biomechanical improvements after CXL, which may be explained by the ultrastructural changes that lead to KC stabilization, as these changes do not alter corneal viscoelasticity [25].

Given the small number of cases in each study group, to perform a statistically significant analysis, correlations were evaluated across the entire cohort of cases. Several correlations were observed between the degree of improvement in corneal parameters and the initial characteristics of the patients [26]. The decrease in spherical equivalent was more pronounced in cases with higher initial values regarding the cylindrical components of refraction and corneal dioptric values. The CRF difference was correlated with the initial cylindrical value, and the Kmax difference was associated with the CRF, SE, and baseline Kmax and Kmin [27]. According to the statistical analysis, the improvement of the corneal resistance factor was positively correlated with the improvement of the corneal dioptric value on the most refractive meridian because of the histopathological changes induced by crosslinking, namely the increase in the diameter of the collagen fibers and the formation of new connections between them [23]. A multivariate analysis identified high baseline SE and Kmax as predictors of significant Kmax flattening [28].

In terms of procedural safety, the corneal thickness of all patients in our study was greater than 400 microns, a value that prevents UVA toxicity to the corneal endothelium [2]. Anterior segment optical coherence tomography (OCT) is crucial for assessing corneal thickness and determining the depth of the demarcation line, which appears as a hyperreflective band in the mid-stroma [29]. In the current study, the demarcation line was identified at an average depth of 278.90 micrometers in the accelerated group and at 280.43 micrometers in the standard group. The demarcation line was detected at a greater depth in cases with lower corneal dioptric power values.

## Conclusions

In this comparative study, CXL was found to be efficient in halting the progression of keratoconus using both the accelerated and standard methods. There were no statistically significant differences in the magnitude of improvement in SE, CCT, corneal diopters values, CH, CRF, or the depth of the demarcation line. However, accelerated CXL is faster and more comfortable for patients and has similar efficiency to standard CXL.

## Data Availability

The datasets generated during and/or analyzed during the current study are available from the corresponding author on reasonable request.

## References

[1] Burcel MG, Corbu C, Coviltir V, Potop V, Constantin M, Dascalescu D (2019). Corneal crosslinking in progressive keratoconus: Comparison of dextran-based and hydroxypropyl methylcellulose-based riboflavin solutions-differences in demarcation line depth and 1 year outcomes. Rev Chim.

[2] Ghaffari R, Hashemi H, Asghari S (2019). Intraoperative OCT for Monitoring Corneal Pachymetry during Corneal Collagen Cross-Linking for Keratoconus. In: A Practical Guide to Clinical Application of OCT in Ophthalmology. IntechOpen.

[3] Hashemi H, Heydarian S, Hooshmand E, Saatchi M, Yekta A, Aghamirsalim M (2020). The Prevalence and Risk Factors for Keratoconus: A Systematic Review and Meta-Analysis. Cornea [Internet].

[4] Gomes JAP, Rodrigues PF, Lamazales LL (2022). Keratoconus epidemiology: A review. Saudi Journal of Ophthalmology.

[5] Huang AJ, Tseng SC, Kenyon KR (1989). Paracellular permeability of corneal and conjunctival epithelia. Investigative ophthalmology & visual science [Internet].

[6] Papachristoforou N, Ueno A, Ledwos K, Bartuś J, Nowińska A, Karska-Basta I (2025). A Review of Keratoconus Cross-Linking Treatment Methods. Journal of Clinical Medicine.

[7] Kamaev P, Friedman MD, Sherr E, Muller D (2012). Photochemical Kinetics of Corneal Cross-Linking with Riboflavin. Investigative Ophthalmology & Visual Science.

[8] O’Brart DPS (2016). Riboflavin for corneal cross-linking. Drugs of today.

[9] Hayes S, Morgan SR, O’Brart DP, O’Brart N, Meek KM (2015). A study of stromal riboflavin absorption in ex vivo porcine corneas using new and existing delivery protocols for corneal cross-linking. Acta Ophthalmologica.

[10] Samaras K, O’Brart D, Doutch J, Hayes S, Marshall J, Meek KM (2009). Effect of Epithelial Retention and Removal on Riboflavin Absorption in Porcine Corneas. Journal of Refractive Surgery.

[11] Seiler TG, Ehmke T, Fischinger I, Zapp D, Stachs O, Seiler T (2015). Two-Photon Fluorescence Microscopy for Determination of the Riboflavin Concentration in the Anterior Corneal Stroma When Using the Dresden Protocol. Invest Ophthalmol Vis Sci.

[12] Giannaccare G, Murano G, Carnevali A, Yu AC, Vaccaro S, Scuteri G (2021). Comparison of Amsler-Krumeich and Sandali Classifications for Staging Eyes with Keratoconus. Applied Sciences.

[13] Salmon JF (2019). Kanski’s Clinical Ophthalmology E-Book: A Systematic Approach.

[14] Salman AM, Darwish TR, Haddad YH, Shabaan RH, Askar MZ (2021). Accelerated versus Standard Corneal Cross-linking for Progressive Keratoconus in Syria. Journal of Ophthalmic & Vision Research.

[15] Polido J, dos Xavier Santos Araújo ME, Alexander JG, Cabral T, Ambrósio R, Freitas D (2022). Pediatric Crosslinking: Current Protocols and Approach. Ophthalmology and Therapy.

[16] Iqbal M, Elmassry A, Saad H, Am Gad A, Ibrahim O, Hamed N (2020). Standard cross-linking protocol versus accelerated and transepithelial cross-linking protocols for treatment of paediatric keratoconus: a 2-year comparative study. Acta Ophthalmol.

[17] Li Y, Lu Y, Du K, Yin Y, Hu T, Fu Y (2022). Comparison of Efficacy and Safety Between Standard. Accelerated Epithelium-Off and Transepithelial Corneal Collagen Crosslinking in Pediatric Keratoconus: A Meta-Analysis. Front Med.

[18] Kandel H, Abbondanza M, Gupta A, Mills R, Watson AS, Petsoglou C (2023). Comparison of standard versus accelerated corneal collagen cross-linking for keratoconus: 5-year outcomes from the Save Sight Keratoconus Registry. Eye.

[19] Burcel MG, Lacraru IC, Dascalescu DMC, Corbu MC, Potop V, Coviltir V (2022). Assessment of two-year clinical outcomes after keratoconus treatment using two different crosslinking protocols. Eur Rev Med Pharmacol Sci.

[20] Van L, Bennett S, Nicholas SE, Hjortdal J, McKay TB, Karamichos D (2023). Prospective Observational Study Evaluating Systemic Hormones and Corneal Crosslinking Effects in Keratoconus. Ophthalmology Science.

[21] Hamid A, Jahadi-Hosseini H, Khalili MR, Jahanbani-Ardakani H (2023). Corneal Biomechanical Changes after Corneal Cross-Linking in Patients with Keratoconus. Journal of Current Ophthalmology.

[22] Wollensak G, Spoerl E, Seiler T (2003). Stress-strain measurements of human and porcine corneas after riboflavin-ultraviolet-A-induced cross-linking. Journal of cataract and refractive surgery [Internet].

[23] Wollensak G, Wilsch M, Spoerl E, Seiler T (2004). Collagen fiber diameter in the rabbit cornea after collagen crosslinking by riboflavin/UVA. Cornea [Internet].

[24] Woo JH, Iyer JV, Lim L, Htoon Hla M, Mehta JS, Chan CML (2017). Conventional Versus Accelerated Collagen Cross-Linking for Keratoconus: A Comparison of Visual Refractive, Topographic and Biomechanical Outcomes. The Open Ophthalmology Journal.

[25] De Bernardo M, Capasso L, Lanza M, Tortori A, Iaccarino S, Cennamo M (2014). Long-term results of corneal collagen crosslinking for progressive keratoconus. Journal of Optometry.

[26] Faramarzi A, Hassanpour K, Rahmani B, Yazdani S, Kheiri B, Sadoughi MM (2021). Systemic supplemental oxygen therapy during accelerated corneal crosslinking for progressive keratoconus: randomized clinical trial. Journal of cataract and refractive surgery [Internet].

[27] Toprak I, Yaylalı V, Yildirim C (2014). Factors affecting outcomes of corneal collagen crosslinking treatment. Eye (London; England) [Internet].

[28] Wajnsztajn D, Shmueli O, Zur K, Frucht-Pery J, Solomon A (2022). Predicting factors for the efficacy of cross-linking for keratoconus. PLoS ONE.

[29] Roongpoovapatr V, Shousha MA, Charukamnoetkanok P (2020). Keratoconus Treatment Toolbox: An Update. In: Eyesight and Imaging - Advances and New Perspectives. IntechOpen.

